# Identification and Validation of a Four-Long Non-coding RNA Signature Associated With Immune Infiltration and Prognosis in Colon Cancer

**DOI:** 10.3389/fgene.2021.671128

**Published:** 2021-07-05

**Authors:** Yanbo Wang, Jing Liu, Fenghai Ren, Yanjie Chu, Binbin Cui

**Affiliations:** ^1^Department of Thoracic Surgery, Harbin Medical University Cancer Hospital, Harbin, China; ^2^Department of Gastroenterology and Hepatology, The Second Affiliated Hospital of Harbin Medical University, Harbin, China; ^3^Department of Colorectal Surgery, Harbin Medical University Cancer Hospital, Harbin, China

**Keywords:** long non-coding RNAs, colon cancer, immune microenvironment, tumor immune infiltration, integrative analysis

## Abstract

The emerging evidence has demonstrated the critical roles of long non-coding RNAs (lncRNAs) as regulators in the tumor immune microenvironment (TIME). However, the tumor immune infiltration-associated lncRNAs and their clinical significance in colon cancer have not yet been thoroughly investigated. This study performed an integrative analysis of lncRNA expression profiles and immune cell infiltration profiles and identified 258 immune infiltration-associated lncRNAs. Of them, four lncRNAs (AC008494.3, LINC00926, AC022034.1, and SNHG26) were significantly and independently associated with the patient’s overall survival. Finally, we developed a tumor immune infiltration-associated lncRNA signature (TIILncSig) comprising of these four lncRNAs, which can divide colon cancer patients of The Cancer Genome Atlas (TCGA) into high-risk and low-risk groups with a significantly different outcome [Hazard ratio (HR) = 2.718, 95% CI = 1.955–3.779, *p* < 0.001]. Prognostic performance of the TIILncSig was further validated in another independent colon cancer cohort (HR = 1.832, 95% CI = 1.045–3.21, *p* = 0.034). Results of multivariate Cox regression and stratification analysis demonstrated that the TIILncSig is an independent predictive factor from other clinical features (HR = 2.687, 95% CI = 1.912–3.776, *p* < 0.001 for TCGA cohort and HR = 1.837, 95% CI = 1.047–3.223, *p* = 0.034 for GSE17538 cohort). Literature analysis provided experimental evidence supporting roles of the TIILncSig in cancer carcinogenesis and progression and immune regulation. Summary, our study will help to understand the mechanisms of lncRNAs in immune regulation in the tumor microenvironment and provide novel biomarkers or targets for prognosis prediction and therapy decision-making for patients with colon cancer.

## Introduction

Colorectal cancer (CRC) is the most common type of gastrointestinal cancer and is the second leading cause of cancer-related death (Siegel et al., 2020a,b). CRC cases were classified into colon tumors and rectum tumors according to codes from the International Classification of Diseases for Oncology (Siegel et al., 2020a). It is estimated to be 104,610 individuals newly diagnosed with colon cancer in the United States in 2020 (Siegel et al., 2020a). The tumor, lymph node, metastasis (TNM) staging system, histological differentiation grade, and tumor sidedness have been widely used for colon cancer classification, prognosis prediction, and therapy decision-making. Surgical removal of the primary tumor followed by adjuvant chemotherapy is the main treatment for colon cancer patients. However, in clinical practice, the TNM staging system was not enough to predict prognosis and make therapeutic decisions for the patient with colon cancer patients. There is growing evidence that molecular biomarkers have become indispensable in the more personalized precision medicine era (Vacante et al., 2018; Koncina et al., 2020).

Increasing evidence has suggested that the tumor immune microenvironment (TIME) plays either tumor-promoting or tumor-suppressive roles in various cancers and profoundly influences tumor prognosis and therapy response (Chew et al., 2012; Binnewies et al., 2018). The tumor microenvironment of CRC has been characterized using different methods in several studies and highlighted its prognostic, predictive, and therapeutic implications. For example, tumor-infiltrating lymphocytes (TILs), an essential component of TIME, have been recognized as an essential histopathologic feature associated with the prognosis of patients with colorectal cancer (Idos et al., 2020). Recent studies have found that transcriptomic or epigenetic features are associated with specific immune cell subpopulations of TIME. They, therefore, could be used to infer the composition of tumor-infiltrating immune cells (Zhang et al., 2020b). Long non-coding RNAs, constituting the major class of non-coding RNAs (ncRNAs), have been recognized as an essential regulator involved in nearly all biological progress. The emerging roles of lncRNAs as essential regulators of the human immune system have been recognized in recent studies (Turner et al., 2014; Chen et al., 2017). Increasing evidence suggested that lncRNAs played vital roles in the development, differentiation, activation of different immune cells and contributed to the modulation of innate and adaptive immunity. Furthermore, lncRNAs could act as communicators and mediators between the tumor microenvironment and cancer cells, highlighting their potential as immunotherapy targets and biomarkers. For example, Sun et al. (2020) identified 57 tumor immune infiltration-associated lncRNAs in non-small cell lung cancer and found that seven of them are associated with patient’s survival and response to immune checkpoint inhibitor (ICI) immunotherapy. Zhou et al. (2020) developed a lncRNA signature of tumor-infiltrating B lymphocytes that are predictive of prognosis and immunotherapy response in bladder cancer. Although continued efforts are coming, the tumor immune infiltration-associated lncRNAs and their clinical significance in colon cancer have not yet been thoroughly investigated.

In this study, we performed an integrative analysis of lncRNA expression profiles, immune cell infiltration profiles, and clinical profiles to infer tumor immune infiltration-associated lncRNAs in colon cancer and explored their value in predicting prognosis.

## Materials and Methods

### Clinical and Transcriptomic Data of Colon Cancer Patients

Clinical and transcriptomic data of 512 colon cancer patients were obtained from UCSC Xena Browser (GDC TCGA Colon Cancer cohort).^1^ The RNA-seq gene expression level 3 data [log2(RPKM +1) transformed] were used for this study. After removed those patients with overall survival <30 days, a total of 419 was left for further study. Clinical and transcriptomic data of 219 colon cancer patients were derived from Gene Expression Omnibus (GEO) database.^2^ Microarray data profiled by Affymetrix Human Genome U133 Plus 2.0 Array was used and then background correction, quantile normalization using the R package “affy.”

### Acquisitions of lncRNA Expression Profiles of Colon Cancer Patients

Approved human lncRNA information was obtained from HUGO Gene Nomenclature Committee (HGNC).^3^ After cross-referenced and removed lncRNAs with 0 value in more than 20% of samples, a total of 3,959 lncRNAs were retained for RNA-seq data. For microarray data, a total of 5,919 lncRNAs were retained through repurposing array probes into the human genome and HGNC database. After cross-validation analysis among different cohorts based on different platforms, 2,235 overlapped lncRNA among different platforms were kept for further analysis.

### Calculation of Infiltrating Immune Cell Abundance in the Tumor Microenvironment

The relative proportions of 22 types of infiltrating immune cells in the tumor microenvironment were inferred using the CIBERSORT algorithm^4^ with the default signature matrix at 500 permutations (Newman et al., 2015).

### Statistical Analysis

The association between lncRNA expression and the abundance of infiltrating immune cells was measured using the Pearson correlation coefficient (PCC). The association between lncRNA expression and overall survival were evaluated using the univariate and multivariate Cox regression analysis. The lncRNA-based scoring model was developed using the linear combination of lncRNA biomarkers’ transformed expression values with the multivariate Cox regression coefficient as the weight. The differences in overall survival between the high-risk and low-risk groups were assessed using the Kaplan-Meier survival curves and log-rank tests. Hazard ratios (HR) and 95% CI were calculated. The time-dependent receiver operating characteristic (ROC) curve and area under the time-dependent ROC curve (AUC) was calculated for assessing the predictive performance for survival at 3- and 5-years. All statistical analyses were conducted with R software and Bio-conductor (version 3.6.3).

## Results

### Identification of Prognostic Tumor Immune Infiltration-Associated lncRNAs in Colon Cancer

**Figure 1:**
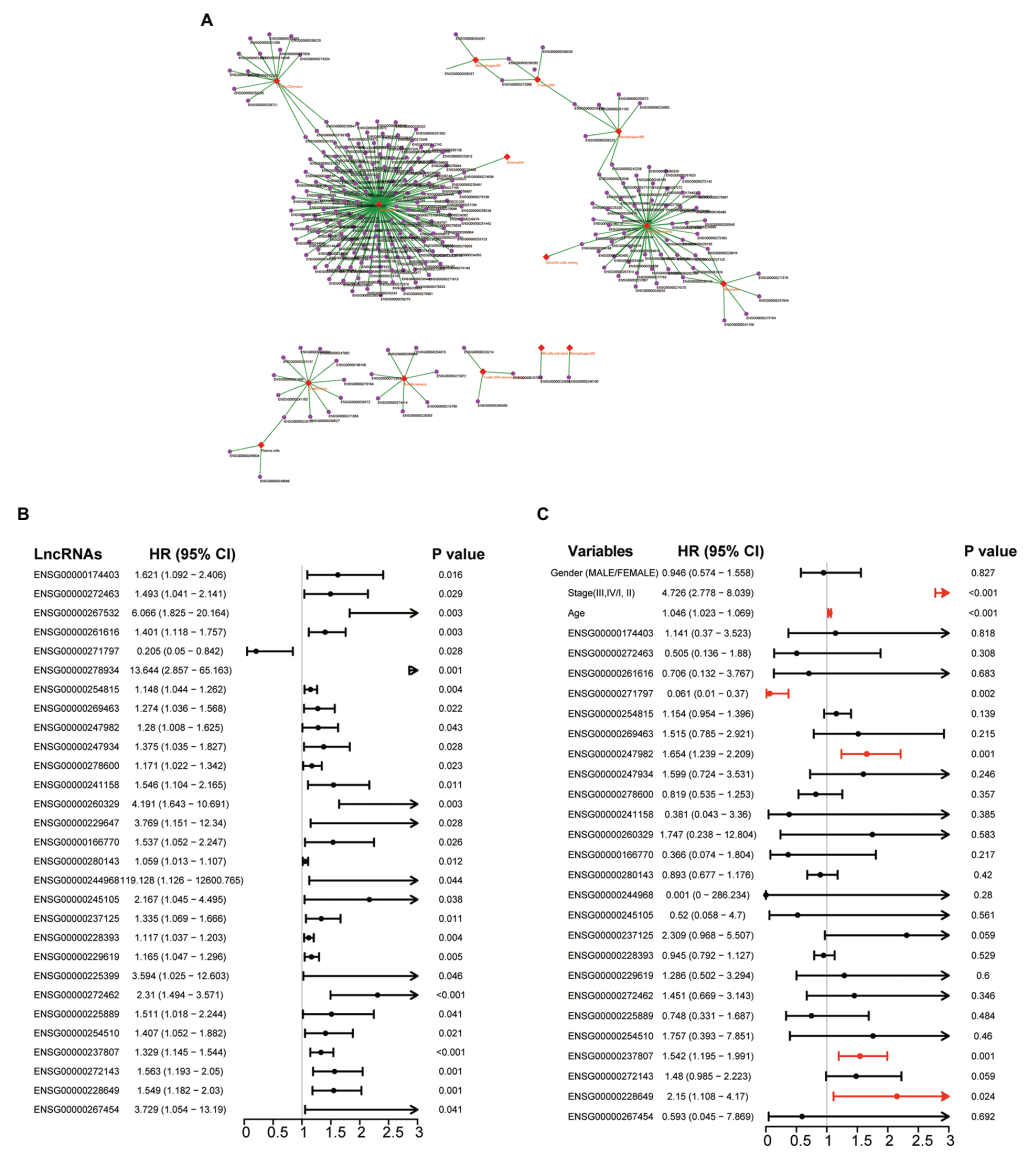
Identification of prognostic tumor immune infiltration-associated long non-coding RNAs (lncRNAs) in colon cancer. **(A)** Network representing the association between lncRNA and infiltrating immune cells using the Cytoscape software. **(B)** Forest plot representing hazard ratio (HR) and 95% CI of each prognostic lncRNAs derived from Univariate analyses with Cox proportional hazards regression. **(C)** Forest plot representing HR and 95% CI of each prognostic lncRNAs derived from multivariate analyses with Cox proportional hazards regression.

We first inferred infiltrating levels of 22 immune cell types in the tumor microenvironment through the CIBERSORT algorithm based on the RNA-seq data of patients in the TCGA cohort. Then, we measured the association of lncRNAs with different infiltrating immune cells by calculating the PCC between expression levels of each lncRNA and infiltrating abundance of each infiltrating immune cells in the TME. Finally, we identified 258 lncRNAs that are highly correlated with infiltrating abundance of at least one of 22 immune cell types (Supplementary File 1), as shown in Figure 1A. To determine the prognostic value of these 258 immune infiltration-associated lncRNAs, we performed univariate Cox regression analysis to examine the association between expression levels of these 258 immune infiltration-associated lncRNAs and overall survival of patients in the TCGA cohort, and found that 29 of 258 immune infiltration-associated lncRNAs are significantly associated with overall survival as shown in Figure 1B. To further investigate the independence of these 29 prognostic immune infiltration-associated lncRNAs in predicting overall survival, we then conducted multivariate Cox regression analysis on the expression level of 29 prognostic immune infiltration-associated lncRNAs with overall survival as a dependent variable and other individual clinical features as explanatory variables, and identified four of 29 prognostic immune infiltration-associated lncRNAs as independent prognostic factors that still maintained a significant association with overall survival after adjusted by other clinical features as shown in Figure 1C (Table 1).

**Table 1:** The detailed information of four prognostic tumor immune infiltration-associated lncRNAs in colon cancer.

Ensemble ID	Gene name	Genomic location	HR	95% CI	Coef	*p*-value
ENSG00000271797	AC008494.3	Chr5: 115,262,505-115,263,448(+)	0.205	0.05–0.842	−1.584	0.028
ENSG00000247982	LINC00926	Chr15: 57,300,365-57,307,769(+)	1.28	1.008–1.625	0.247	0.043
ENSG00000237807	AC022034.1	Ch8: 53,493,523-53,524,336(−)	1.329	1.145–1.544	0.285	<0.001
ENSG00000228649	SNHG26	Chr7: 22,854,126-22,872,945(+)	1.549	1.182–2.03	0.438	0.001

### Development and Evaluation of a Tumor Immune Infiltration-Associated lncRNA Signature in Predicting Survival

**Figure 2:**
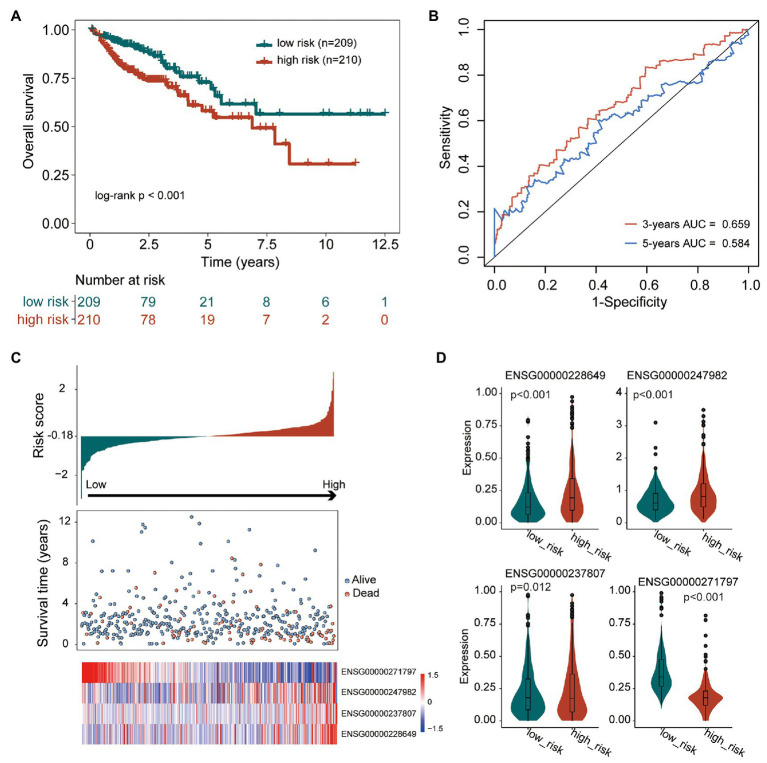
Performance evaluation of the tumor immune infiltration-associated lncRNA signature (TIILncSig) for predicting overall survival in the TCGA cohort. **(A)** Kaplan-Meier survival curves of overall survival between high- and low-risk groups. **(B)** The time-dependent receiver operating characteristic (ROC) analysis of the sensitivity and specificity for survival prediction by the TIILncSig. **(C)** The risk score distribution, survival status, and expression pattern of the TIILncSig. **(D)** Boxplot representing expression differences of lncRNAs between high- and low-risk groups.

To construct a tumor immune infiltration-associated lncRNA signature to predict overall survival, we fitted these four independent prognostic lncRNAs in a multivariate Cox regression model with overall survival as a dependent variable to measure relative contributions. We then developed a tumor immune infiltration-associated lncRNA signature (TIILncSig) by a linear combination of expression levels of these four independent prognostic lncRNAs, weighted by the corresponding coefficient derived from above multivariate analysis according to previous studies, as follows: TIILncSig = expression(AC008494.3)∗(−2.334119617)+expression(LINC00926)∗0.289309684+expression(AC022034.1)∗0.283336978+expression(SNHG26)∗0.579001809. Then patients of the TCGA cohort were assigned a risk score based on TIILncSig and subsequently were classified into high-risk groups (*n* = 210) and low-risk groups (*n* = 209) according to the median value of risk score. As shown in Figure 2A, patients in the high-risk group were observed to have significantly poor overall survival than those in the low-risk group (log-rank test *p* < 0.001; Figure 2A). The three- and five-survival rates of patients in the high-risk group were 73.9 and 58.1%, respectively, whereas the corresponding rates of patients in the low-risk were 86.5 and 72.9%, respectively. Furthermore, the TIILncSig achieved AUC values of 0.659 and 0.584 in predicting 3 and 5-years survival (Figure 2B). The distribution of TIILncSig-based risk score, the survival status and expression pattern of prognostic lncRNAs of patients in the TCGA cohort were plotted. As shown in Figure 2C, more deaths tended to be enriched in the high-risk group relative to the low-risk group. Moreover, AC008494.3 tended to be a protective factor whose high expression is associated with low risk. On the contrary, the other three lncRNAs (LINC00926, AC022034.1, and SNHG26) tended to be risk factors whose high expression is associated with high risk. As shown in Figure 2D, three risky lncRNAs (LINC00926, AC022034.1, and SNHG26) revealed significantly higher expression in the high-risk groups compared to in the low-risk group, and one protective lncRNA AC008494.3was observed to be expressed at low levels in the high-risk group compared to in the low-risk group (Figure 2D).

### Independent Validation of the Prognostic Performance of the TIILncSig

**Figure 3:**
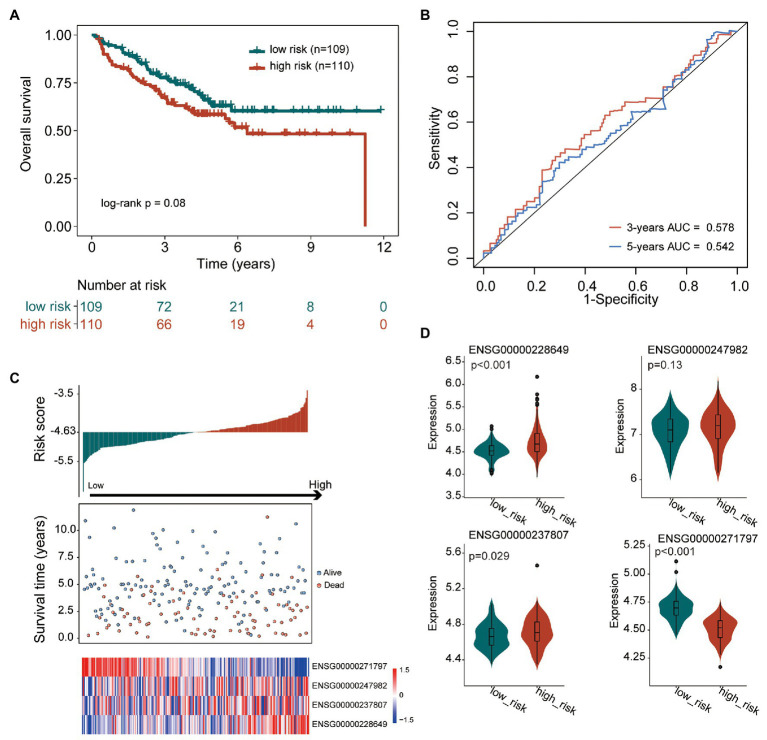
Performance validation of the TIILncSig for predicting overall survival in the GSE17538 cohort. **(A)** Kaplan-Meier survival curves of overall survival between high- and low-risk groups. **(B)** The time-dependent ROC analysis of the sensitivity and specificity for survival prediction by the TIILncSig. **(C)** The risk score distribution, survival status, and expression pattern of the TIILncSig. **(D)** Boxplot representing expression differences of lncRNAs between high- and low-risk groups.

To further confirm the robustness and reliability of the TIILncSig in predicting survival, we further applied the TIILncSig into another independent GEO COAD cohort (GSE17538). The TIILncSig classified 219 patients of the GSE17538 cohort into the high-risk group (*n* = 110) and low-risk group (*n* = 109) with obvious different overall survival (log-rank test *p* = 0.08; Figure 3A). As shown in Figure 3A, the overall survival of patients in the high-risk group patients was shorter than that of patients in the low-risk group patients. The three- and five-survival rates of patients in the high-risk group were 67.3 and 58.4%, respectively, whereas the corresponding rates of patients in the low-risk group were 77.7 and 63.0%. Furthermore, the TIILncSig achieved AUC values of 0.578 and 0.542 in predicting 3 and 5-years survival (Figure 3B). The distribution of TIILncSig-based risk score, the survival status and expression pattern of prognostic lncRNAs of patients in the independent GSE17538 cohort were similar to those observed in the TCGA cohort. As shown in Figures 3C,D, higher expression of three risky lncRNAs (LINC00926, AC022034.1, and SNHG26) and a lower expression of one protective lncRNA (AC008494.3) were observed in patients of the high-risk group compared to those in the low-risk group.

### Independence of the TIILncSig From Other Clinical Features

**Figure 4:**
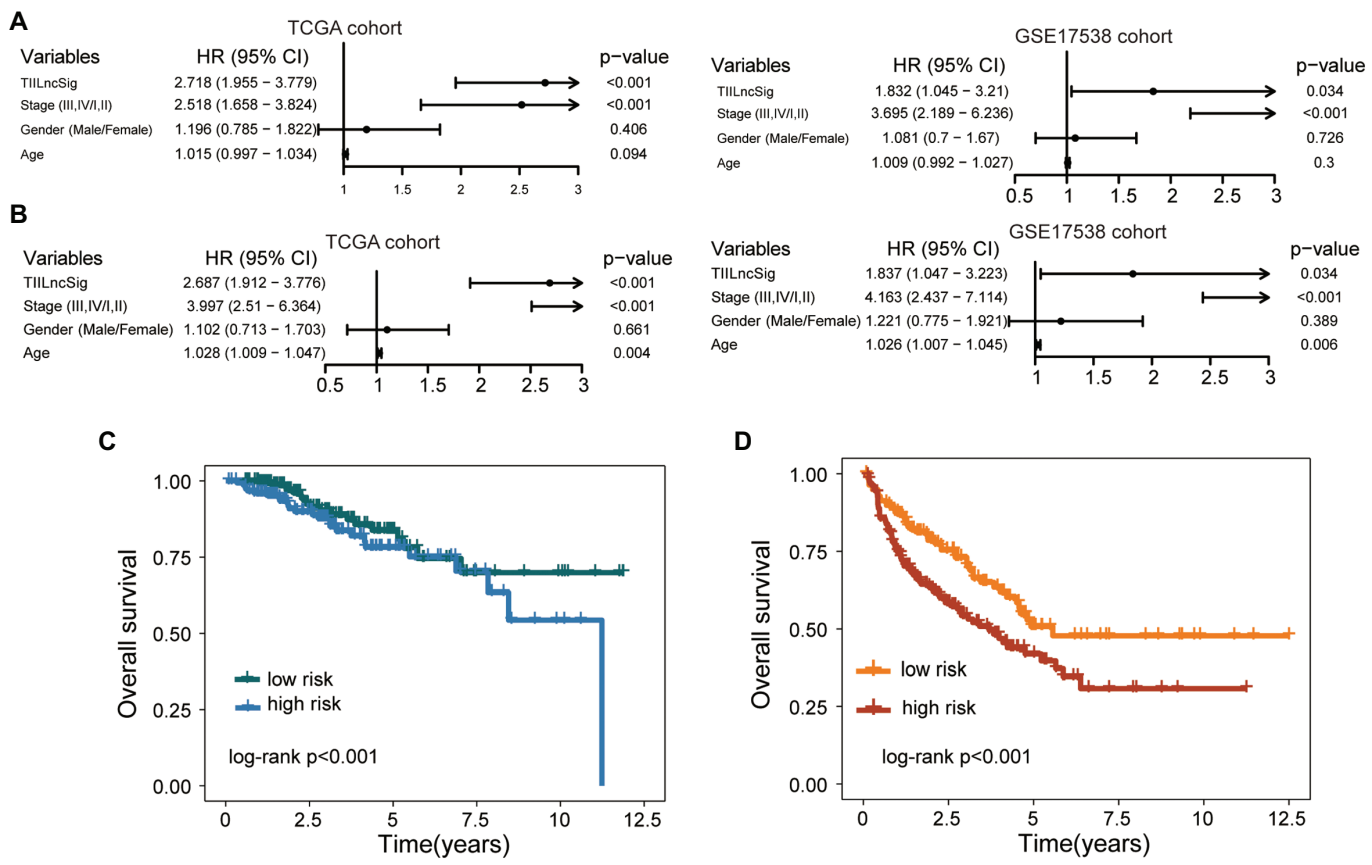
Independence of the TIILncSig from other clinical features. **(A)** Univariable Cox regression analysis of the TIILncSig and other clinical features with overall survival in each cohort. **(B)** Multivariable Cox regression analysis of the TIILncSig and other clinical features with overall survival in each cohort. **(C)** Kaplan-Meier survival curves of overall survival between high- and low-risk groups for patients with stage I and II. **(D)** Kaplan-Meier survival curves of overall survival between high- and low-risk groups for patients with stage III and IV.

Results of the univariate analysis showed that although the TIILncSig is significant with overall survival in the TCGA cohort (HR = 2.718, 95% CI = 1.955–3.779, *p* < 0.001) and GSE17538 cohort (HR = 1.832, 95% CI = 1.045–3.21, *p* = 0.034), the stage is also significant in the univariate analysis (HR = 2.518, 95% CI = 1.658–3.824, *p* < 0.001 for TCGA cohort HR = 3.695, 95% CI = 2.189–6.236, *p* < 0.001 for GSE17538 cohort; Figure 4A). Therefore, we further performed multivariate Cox regression analyses with overall survival as the dependent variable and the TIILncSig and other clinical features as explanatory variables in each cohort to investigate whether the prognostic performance of the TIILncSig is independent of other important clinical features of COAD patients. As shown in Figure 4B, the TIILncSig still had significant associations with overall survival in the TCGA cohort (HR = 2.687, 95% CI = 1.912–3.776, *p* < 0.001) and GSE17538 cohort (HR = 1.837, 95% CI = 1.047–3.223, *p* = 0.034). We further performed a stratification analysis for stage and examined the prognostic value of the TIILncSig within the early stage or late stage. All patients were classified into the early-stage group (Stage I and II) and late-stage group (Stage III and IV). When the TIILncSig was applied to patients with stage I and II, we found that the TIILncSig could effectively classify patients into a high-risk group and low-risk group with significantly different overall survival (*p* < 0.001, log-rank test; Figure 4C). Similar prediction results were observed for patients in the late-stage group. As shown in Figure 4D, patients with stage III and IV were separated into two risk subgroups with significantly different overall survival (*p* < 0.001, log-rank test; Figure 4D). These multivariate and stratification analysis results demonstrated the independence of the TIILncSig from other clinical features in predicting overall survival.

## Discussion

Colon cancer is a well-known heterogeneous disease characterized by distinct clinical, pathological, molecular features, prognosis, and therapy response (Linnekamp et al., 2015; Zhai et al., 2017). Various molecular characteristics have been widely observed to contribute to colon cancer heterogeneity. Different gene expression patterns have been used to define molecularly distinct subtypes with different clinical behavior (Perez-Villamil et al., 2012; Schlicker et al., 2012; Budinska et al., 2013). MicroRNA expression profiles have also been reported to show different expression patterns associated with those obtained by mRNA expression profiling; therefore, they can classify CRC tumors in the same way as mRNA (Sarver et al., 2009; Slattery et al., 2016). Recently, a new ncRNA molecule termed lncRNAs, also revealed different expression profiles associated with distinct clinical features, prognosis, and therapy response and have been used to defined lncRNA-derived molecular subtype (Chen et al., 2014; Zhou et al., 2018a).

Increasing efforts in colon cancer immunogenomics have shown that the type, density, and location of infiltrating immune cells within a tumor have a significant effect on clinical outcome and subtype classification (Zhang et al., 2018; Catalano et al., 2019; Shen et al., 2019). The functional roles and molecular mechanisms of lncRNAs involved in the regulation of tumor immune microenvironment have been reported in some studies (Zhang et al., 2020a). Some known immune-related lncRNAs have been proven to contribute to tumor initiation, growth, and metastasis and acted as potential biomarkers and targets (Zhou et al., 2017, 2018b). However, the tumor immune infiltration-associated lncRNAs and their clinical significance in colon cancer have not yet been fully investigated. Many studies have shown that cellular composition of immune infiltrates in the tumor could be quantified through traditional microscopy-based, but also could be inferred using immunoinformatics algorithms and computational approaches based on genomic, transcriptomic, or DNA methylation data, which provided a very convenient way to unraveled tumor-immune interactions (Zhang et al., 2020b). Therefore, in this study, we first inferred infiltrating profiles of 22 immune cell types in the tumor microenvironment through the CIBERSORT algorithm based on the RNA-seq data of patients in the TCGA cohort. Then, we performed an integrative analysis of lncRNA expression profiles and immune cell infiltration profiles and identified 258 immune infiltration-associated lncRNAs. Combining survival data and Cox regression analysis, four of 258 immune infiltration-associated lncRNAs was found to be independent prognostic factors associated with overall survival after adjusted by other clinical features. To facilitate clinical application, we constructed an lncRNAs-based scoring model based on the expression levels of these four lncRNAs, and applied this model to different patient cohorts. Results from different cohorts demonstrated that the TIILncSig is not only a robust and reliable prognostic factor, but also in independent of other clinical features.

Although more and more lncRNAs have been discovered and identified using the experimental or computational approaches, only a small fraction of them have well been functionally characterized. Abnormal expression of LINC00926 has been observed in several cancers. For example, Wang et al. (2018) found that overexpression of LINC00926 was observed to be associate with improved overall survival in acute myeloid leukemia. Wu and colleagues found that dysregulated expression of LINC00926 was associated with prostate cancer-related fatigue during localized radiation therapy by constructing mRNA and lncRNA regulatory networks. Functional analysis revealed the association of LINC00926 with inflammatory response and immune response-related biological processes (Ye et al., 2018). Further study of Liang et al. (2020) demonstrated the upregulated expression of LINC00926 in late relapsed Hodgkin lymphoma. Recently, Ma et al. (2020) performed a co-expression analysis of lncRNA and immune genes in breast cancer and found that LINC00926 is positively correlated with TNFRSF13C and CD19. The interaction between LINC00926 and the histone H3K4 methyltransferase, MLL1, was confirmed to lead to elevated proinflammatory cytokines and inflammatory states (Bam et al., 2019). Another lncRNAs in this TIILncSig, SNHG26, have also been associated with carcinogenesis and clinical outcome in several cancers. For example, SNHG26 has significantly upregulated in bladder urothelial carcinoma and associated with poor survival (Bao et al., 2017). Overexpression of SNHG6 was found to promote cell proliferation and metastasis in clear cell renal cell carcinoma (ccRCC) by interacting with YBX1 (Zhao et al., 2021). These existing experimental evidence further support the predicted TIILncSig in cancer carcinogenesis and progression and immune regulation. The other two lncRNAs have not been reported to associate with immune progress. Therefore they may be needed to be experimentally and functionally investigated. Although, our results uncovered TIILncSig as a potentially useful biomarker, the TIILncSig would need to be validated in a prospective immunotherapy clinical trial as a companion science trial. Furthermore, whether or not the TIILncSig would be useful in other cancers also should be tested. Finally, our study lacks a validation process for the TIILncSig. Further biological experiments should be made to elucidate the function of the TIILncSig.

In conclusion, we identified several novel lncRNAs involved in the regulation of tumor immune microenvironment associated with different patient clinical outcomes by performing integrative analysis of lncRNA expression profiles, immune cell infiltration profiles, and clinical profiles. Our study will help in understanding the mechanisms of lncRNA in immune regulation in the tumor microenvironment and provide novel biomarkers or targets for prognosis prediction and therapy decision-making for patients with colon cancer.

## Data Availability Statement

Publicly available datasets were analyzed in this study. This data can be found at: clinical and transcriptome data of colon cancer patients was downloaded from UCSC Xena Browser (http://xena.ucsc.edu/, GDC TCGA Colon Cancer cohort) and Gene Expression Omnibus (https://www.ncbi.nlm.nih.gov/geo/query/acc.cgi?acc=GSE17536).

## Author Contributions

BC conceived and designed the experiments. YW, JL, FR, and YC performed the experiments and analyzed the data. YW and JL wrote the paper. All authors contributed to the article and approved the submitted version.

### Conflict of Interest

The authors declare that the research was conducted in the absence of any commercial or financial relationships that could be construed as a potential conflict of interest.

## Footnotes

^1^http://xena.ucsc.edu/

^2^https://www.ncbi.nlm.nih.gov/geo/query/acc.cgi?acc=GSE17536

^3^https://www.genenames.org/

^4^http://cibersort.stanford.edu/
